# Pre-injury frailty and clinical care trajectory of older adults with trauma injuries: A retrospective cohort analysis of A large level I US trauma center

**DOI:** 10.1371/journal.pone.0317305

**Published:** 2025-02-05

**Authors:** Oluwaseun Adeyemi, Corita Grudzen, Charles DiMaggio, Ian Wittman, Ana Velez-Rosborough, Mauricio Arcila-Mesa, Allison Cuthel, Helen Poracky, Polina Meyman, Joshua Chodosh

**Affiliations:** 1 Ronald O Perelman Department of Emergency Medicine, New York University Grossman School of Medicine, New York, NY, United States of America; 2 Department of Medicine, Memorial Sloan Kettering Cancer Center, New York, NY, United States of America; 3 Department of Surgery, New York University Grossman School of Medicine, New York, NY, United States of America; 4 Department of Population Health, New York University Grossman School of Medicine, New York, NY, United States of America; 5 Department of Medicine, New York University School of Medicine, New York, NY, United States of America; 6 Department of Trauma, New York University Grossman School of Medicine, New York, NY, United States of America; 7 Medicine Service, Veterans Affairs New York Harbor Healthcare System, New York, NY, United States of America; Policlinico Universitario A. Gemelli IRCCS - Universita Cattolica del Sacro Cuore Roma, ITALY

## Abstract

**Background:**

Pre-injury frailty among older adults with trauma injuries is a predictor of increased morbidity and mortality.

**Objectives:**

We sought to determine the relationship between frailty status and the care trajectories of older adult patients who underwent frailty screening in the emergency department (ED).

**Methods:**

Using a retrospective cohort design, we pooled trauma data from a single institutional trauma database from August 2020 to June 2023. We limited the data to adults 65 years and older, who had trauma injuries and frailty screening at ED presentation (N = 2,862). The predictor variable was frailty status, measured as either robust (score 0), pre-frail (score 1–2), or frail (score 3–5) using the FRAIL index. The outcome variables were measures of clinical care trajectory: trauma team activation, inpatient admission, ED discharge, length of hospital stay, in-hospital death, home discharge, and discharge to rehabilitation. We controlled for age, sex, race/ethnicity, health insurance type, body mass index, Charlson Comorbidity Index, injury type and severity, and Glasgow Coma Scale score. We performed multivariable logistic and quantile regressions to measure the influence of frailty on post-trauma care trajectories.

**Results:**

The mean (SD) age of the study population was 80 (8.9) years, and the population was predominantly female (64%) and non-Hispanic White (60%). Compared to those classified as robust, those categorized as frail had 2.5 (95% CI: 1.86–3.23), 3.1 (95% CI: 2.28–4.12), and 0.3 (95% CI: 0.23–0.42) times the adjusted odds of trauma team activation, inpatient admission, and ED discharge, respectively. Also, those classified as frail had significantly longer lengths of hospital stay as well as 3.7 (1.07–12.62), 0.4 (0.28–0.47), and 2.2 (95% CI: 1.71–2.91) times the odds of in-hospital death, home discharge, and discharge to rehabilitation, respectively.

**Conclusion:**

Pre-injury frailty is a predictor of clinical care trajectories for older adults with trauma injuries.

## Introduction

Frailty is a clinical syndrome comprising weakness, slowness, diminished physical activities, exhaustion, and weight loss [1, 2]. It is a chronically acquired clinical state that manifests with increased vulnerability to dependency and disability when exposed to physiological and external stressors [3, 4]. It is estimated that 10 to 15% of community-dwelling older adults are frail [5, 6], and among older adults with trauma injuries, pre-injury frailty prevalence ranges from 2% to 33% [7]. Frailty not only predisposes older adults to injuries such as falls [8, 9] but also increases injury-associated morbidity and mortality [7]. While the natural history of frailty includes the potential for reversal and improvement [4], frailty tends to progress more during acute stress conditions such as traumatic injuries [10]. The inability to mount an adequate physiologic response to trauma leads to worsening weakness, weight loss, and diminished physical activities, which culminate in the loss of one or more domains of activities of daily living and disability [5, 11].

Earlier studies have reported the association between pre-injury frailty and morbidity and mortality among older adults [12–14]. However, little is known about the role pre-injury frailty plays in the clinical care trajectory among older adults with trauma injuries. Earlier studies have reported that hospitalized older adults spend more than 80 percent of their hospital stay lying in bed [15, 16], and approximately 20 percent lose their ability to walk unassisted at discharge [17]. Sarcopenia, a pathologic feature of physical frailty and clinically manifested as loss of muscle mass [18], develops as early as within the first 72 hours of patient admission [15]. It is, therefore, possible that without an intent to manage frailty as a comorbid illness, pre-injury frailty and/or frailty progression may influence the clinical care trajectory of older adults with trauma injuries.

While frailty cannot be corrected during a single inpatient admission, it can be managed. Identifying and managing pre-injury frailty among older adults with trauma injuries can aid in slowing down clinical frailty progression through early initiation of nutritional rehabilitation, exercise physiotherapy, and early mobilization [10, 19]. We hypothesized that, in the absence of deliberate interventions to manage frailty during an index hospital stay, injured older adults with pre-injury frailty who present at the emergency department (ED) will be more likely to receive higher levels of trauma care, admitted to inpatient units, have longer hospital stays, and more likely to die during index hospital admission. This study, therefore, aims to assess the association between pre-injury frailty and the clinical care trajectory experienced by older adults with trauma injuries during an index ED visit.

## Methods

### Study de sign and population

For this retrospective cohort study, we pooled trauma data from the institutional trauma registry of a large urban level I trauma center that serves a racially and ethnically diverse population. The study population was older adults with trauma injuries who presented to the ED between August 2020 and June 2023. This study is among the studies focused on exploring the diagnostic accuracies and clinical relevance of a novel scoring tool for older adults with trauma injuries. We obtained Institutional Review Board (IRB) approval from the New York University Langone Health IRB (i20_01316_MOD05). De-identified data were extracted from the institutional trauma database between July and December 2023 by the medical record staff at the trauma department. We had no access to information that could identify individual participants after data collection. The results we present follow the Strengthening the Reporting of Observational Studies in Epidemiology guidelines [20].

### Inclusion and exclusion criteria

Between August 2020 and June 2023, there were a total of 3,093 ED visits by older adults who sustained trauma injuries. A total of 199 older adults had 231 multiple visits (two or more) to the ED. We retained the most recent of these visits and excluded the older visits. Hence the final sample size was 2,862 older adults with trauma injuries.

### Outcome variable: Clinical care trajectory

We measured clinical care trajectory using seven variables: (1) trauma team activation, (2) inpatient admission, (3) ED discharge, (4) length of hospital stay, (5) in-hospital death, (6) home discharge, and (7) discharge to rehabilitation. The first three outcome variables represent events in the ED, while the last four variables represent events during the hospital stay. Trauma team activation was defined as a binary variable (1/0), with 1 representing Level I or II activations and 0 representing consults or those with traumatic injury but not leveled. Level I activations involved a full trauma team response, which may include Neurosurgery, Orthopedics, Anesthesia, and other sub-specialties, while Level II involved a more limited response involving the trauma attendings, physician assistants, radiology, and ED physicians. In the trauma registry, ED discharge disposition was coded as admitted, died, discharged home, or referred/transferred to another hospital. We, therefore, defined inpatient admission as an ED disposition coded as admitted–those relocated to an inpatient unit, and ED discharge as ED disposition coded as discharged home.

Length of hospital stay was defined as the duration (in days) from inpatient admission to the time of discharge order from the index hospital or transfer order to another level I hospital. Patients who were discharged from the ED were assigned a value of zero for the in-hospital length of stay. In the trauma dataset, hospital discharge disposition was coded as death, discharged home with or without home health services, left against medical advice, discharged to hospice, discharged to rehabilitation or skilled nursing facility, and transferred to another acute care hospital. In-hospital death was defined as a binary variable (1/0), with 1 representing persons who died either in the ED or during hospital admission. We defined home discharge as a hospital disposition coded as discharged home with or without home health services. We defined discharge to rehabilitation as hospital disposition coded as discharged to rehabilitation or skilled nursing facility, i.e., any patient transfer to acute or subacute inpatient rehabilitation, new placement in a skilled nursing facility, or return to a skilled nursing facility. All six measures, except the length of hospital stay, were measured as binary variables. The length of hospital stay was measured as a continuous variable.

### Predictor variable: Frailty

The predictor variable was frailty status, assessed at each patient’s ED presentation. Frailty status was defined using the FRAIL index—an acronym for Fatigue, Resistance, Ambulation, Illnesses, and Loss of Weight [3, 21]. The FRAIL index has a good face and construct validity and reliability of 0.53 [22]. This measure of frailty was selected because it can be rapidly administered and integrated into the workflow in the ED. Each of the five items in the FRAIL index is measured as a binary variable (yes = 1, no = 0). The FRAIL score, therefore, ranges from 0 to 5. Consistent with the FRAIL index scoring, we generated three ordered categories from the scores: Robust (a score of 0), Pre-frail (scores 1 to 2), and Frail (scores 3 to 5) (Table 1) [23, 24].

**Table 1:** Frailty index, categorization, and scoring.

Item Index	Yes = 1, No = 0
**Fatigue: **Are you fatigued?	Yes/No
**Resistance**: Are you unable to walk up 1 flight of stairs?	Yes/No
**Ambulation: **Are you unable to walk 1 block?	Yes/No
**Illnesses**: Do you have more than 5 illnesses?	Yes/No
**Loss of Weight**: Have you lost more than 5% of body weight in the past 6 months?	Yes/No
**Score Range**	0–5
**Categorization**	Score
**Robust**	0
**Pre-frail**	1–2
**Frail**	3–5

FRAIL: An acronym for Fatigue, Resistance, Ambulation, Illnesses, and Loss of weight

### Potential confounders

We controlled for age, sex, race/ethnicity, health insurance type, body mass index, injury mechanism, recurrent fall injury, injury severity, Charlson Comorbidity Index, and the Glasgow Coma Scale at the ED presentation. Age was measured as a continuous variable, while sex was measured as a binary variable. Race/ethnicity was measured as a four-level categorical variable of non-Hispanic White, non-Hispanic Black, Hispanic, and other races. Health insurance type was measured as a three-category nominal variable–Medicare/Medicaid, other health insurance, and no health insurance. Body mass index was measured as an ordered variable of underweight (<18.5 kg/m^2^), normal weight (18.5–24.9 kg/m^2^), overweight (25–29.9 kg/m^2^), and obese (≥ 30.0 kg/m^2^). We defined injury mechanism as either fall or non-fall-related. Recurrent fall injury was measured as a binary variable and defined as the occurrence of ED presentation due to more than one fall-related injury during the study period. We defined the injury severity as a continuous variable using the Injury Severity Scale (ISS) score. The ISS score, computed using the abbreviated injury score of the top three injured body regions, typically ranges from 0 to 75, with 0 representing no injury and 75 representing non-survivable injury [25]. Also, we defined the Charlson Comorbidity index as a four-level ordered variable of none, one, two, and three or more chronic illnesses. The Glasgow Coma Scale score ranges from 3 to 15, and we measured it as a three-level ordered category of mild (13 to 15), moderate (9 to 12), and severe head injury (3 to 8).

### Analysis

A review of our data showed missing values in the following variables: frailty (22.5%), injury severity score (7.2%), body mass index (5.6%), race/ethnicity (1.9%), and Glasgow coma scale score (1.0%). We established that the missingness was at random and performed multiple imputations for missing data, using the multiple imputations with chained equation (MICE) [26, 27]. The MICE model was strengthened by informational variables, which included age, sex, insurance type, injury type, Charlson comorbidity index, ED admission status, triage category, length of stay, in-hospital mortality status, home discharge, and discharge to rehabilitation. We conducted 100 iterations, producing 100 predicted values for all missing data points. We then determined the final value by averaging the predicted values, in line with prior research on multiple imputations [28, 29].

We report summary statistics (mean, standard deviation (SD), median, first and third quartile) and frequency distribution of the selected variables and assess the distribution of variables across the spectrum of robust, pre-frail, and frail categories. Differences across the frailty spectrum were assessed using the Chi-square test, one-way ANOVA, and Kruskal-Wallis test as appropriate. We performed univariable and multivariable logistic regression to assess the relationship between the frailty categories and the measures of clinical care trajectory (excluding length of hospital stay) and reported the odds ratio (OR) and 95% confidence intervals (CI). Also, we performed univariable and multivariable quantile regression to assess the association between the frailty categories and the length of stay and report the median difference (MD) and 95% CI. Lastly, we computed the predicted probabilities of trauma team activation, inpatient admission, ED discharge, in-hospital death, home discharge, discharge to rehabilitation, and the predicted estimates of the lengths of hospital stay. Data were analyzed using STATA version 17 [30].

## Results

The mean (SD) of the sample population was 80 (8.9) years (Table 2). The population was predominantly female (64%) and non-Hispanic White (60%). Approximately 52% had Medicare or Medicaid insurance, and 53% were either overweight (32%) or obese (21%). Falls accounted for 90% of the injuries, 7% had recurrent fall injuries, and 43% of the population had no co-morbid condition. The median (Q1, Q3) injury severity score was 5.0 (2.0, 9.0), and 97% had mild head injuries. Approximately 30% of the patients had trauma teams activated for their care while in the ED. Sixty-two percent had inpatient admission from the ED, while 38% were discharged from the ED. The median (Q1, Q3) length of hospital stay was 2 days (0.0, 5.0), and 1.6% died while on admission. Also, 61% were discharged home, and 33% were discharged to rehabilitation.

**Table 2:** Summary and frequency distribution of the demographic, injury, and measures of care trajectory of older adults with trauma injuries stratified by frailty category (N = 2,862).

Variables	Total Population	Frailty Categories			p-value*
	(N = 2,862)	Robust 940 (32.8)	Pre-Frail 1,222 (42.7)	Frail 700 (24.5)	
Age in years (Mean (SD))	80.3 (8.9)	76.1 (8.1)	81.0 (8.4)	84.8 (8.4)	<0.001**
Sex*					
Female	1,842 (64.4)	591 (62.9)	803 (65.7)	448 (64.0)	0.383
Male	1,020 (35.6)	349 (37.1)	419 (34.3)	252 (36.0)	
Race/Ethnicity					
Non-Hispanic Whites	1,719 (60.0)	492 (52.3)	786 (64.3)	441 (63.0)	<0.001
Non-Hispanic Blacks	94 (3.3)	34 (3.6)	34 (2.8)	26 (3.7)	
Hispanic	471 (16.5)	178 (18.9)	195 (16.0)	98 (14.0)	
Other Races	578 (20.2)	236 (25.1)	207 (16.9)	135 (19.3)	
Insurance Status					
Medicare/Medicaid	1,496 (52.3)	394 (41.9)	664 (54.3)	438 (62.5)	<0.001
Other insurance	1,308 (45.7)	516 (54.9)	541 (44.3)	251 (35.9)	
No insurance	58 (2.0)	30 (3.2)	17 (1.4)	11 (1.6)	
Body Mass Index					
Normal	1,161 (40.6)	361 (38.4)	491 (40.2)	309 (44.1)	<0.001
Underweight	185 (6.4)	44 (4.7)	74 (6.1)	67 (9.6)	
Overweight	924 (32.3)	358 (38.1)	386 (31.6)	180 (25.7)	
Obese	592 (20.7)	177 (18.8)	271 (22.2)	144 (20.6)	
Injury type					
Non-fall injury	279 (9.8)	162 (17.2)	92 (7.5)	25 (3.6)	<0.001
Fall-related injury	2,583 (90.2)	778 (82.8)	1,130 (92.5)	675 (96.4)	
Recurrent fall-related injury					
No	2,672 (93.4)	906 (96.4)	1,137 (93.0)	629 (89.9)	<0.001
Yes	190 (6.6)	34 (3.6)	85 (7.0)	71 (10.1)	
Injury Severity					
Median Score (Q1, Q3)	5.0 (2.0, 9.0)	5.0 (2.0, 9.0)	4.5 (1.0, 9.0)	5.0 (1.0, 9.0)	<0.001
Charlson Comorbidity Index					
No comorbidity	1,218 (42.6)	599 (63.7)	484 (39.6)	135 (19.3)	<0.001
1 comorbidity	1,037 (36.2)	280 (29.8)	487 (39.9)	270 (38.6)	
2 comorbidities	415 (14.5)	52 (5.5)	192 (15.7)	171 (24.4)	
3 or more comorbidities	192 (6.7)	9 (1.0)	59 (4.8)	124 (17.7)	
Glasgow Coma Scale Score					
Mild	2,785 (97.3)	936 (99.6)	1,212 (99.1)	637 (91.0)	<0.001^#^
Moderate	46 (1.6)	2 (0.2)	8 (0.7)	36 (5.1)	
Severe	31 (1.1)	2 (0.2)	2 (0.2)	27 (3.9)	
Trauma Team Activation					
Yes	861 (30.1)	192 (20.4)	388 (31.8)	281 (40.1)	<0.001
No	2,001 (69.9)	748 (79.6)	834 (68.2)	419 (59.9)	
Inpatient Admission					
Yes	1,760 (61.5)	460 (48.9)	748 (61.2)	552 (78.9)	<0.001
No	1,102 (38.5)	480 (51.1)	474 (38.8)	148 (21.1)	
ED Discharge					
Yes	1,086 (38.0)	474 (50.4)	469 (38.4)	143 (20.4)	<0.001
No	1,776 (62.0)	466 (49.6)	753 (61.6)	557 (79.6)	
Length of Hospital Stay					
Median Days (Q1, Q3)	2.0 (0.0, 5.0)	0.0 (0.0, 4.0)	2.0 (0.0, 4.0)	4.0 (1.0, 6.0)	<0.001^##^
Inhospital Death					
Yes	45 (1.6)	4 (0.4)	11 (0.9)	30 (4.3)	<0.001
No	2,817 (98.4)	936 (99.6)	1,211 (99.1)	670 (95.7)	
Home Discharge					
Yes	1,750 (61.2)	699 (74.4)	754 (61.7)	297 (42.4)	<0.001
No	1,112 (38.8)	241 (25.6)	468 (38.3)	403 (57.6)	
Discharge to Rehabilitation					
Yes	936 (32.7)	211 (22.5)	400 (32.7)	325 (46.4)	<0.001
No	1,926 (67.3)	729 (77.5)	822 (67.3)	375 (53.6)	

*: Chi-Square test performed except otherwise specified

**: One way ANOVA performed; #: Fisher’s exact test performed.; ##: Kruskal Wallis test performed

Of the 2,862 patients, 33%, 43%, and 24% were categorized as robust, pre-frail, and frail, respectively (Table 2). The mean age significantly increased from robust to pre-frail and frail categories (p<0.001) and there were significant differences across the frailty categories by race/ethnicity (p<0.001), body mass index (p = 0.001), injury type (p<0.001), recurrent fall injury (p<0.001), injury severity score (p<0.001), Charlson comorbidity index (p<0.001) and Glasgow coma scale score (p<0.001). The proportion of patients who had trauma teams activated for their care increased from 20% to 32% and 40% in the robust, pre-frail, and frail categories, respectively (p<0.001). The proportion of patients who had inpatient admission increased from 49% to 61% and 79% in the robust, pre-frail, and frail categories, respectively (p<0.001). The proportion of those who were discharged from the ED decreased from 50% to 38% and 20% across the three categories (p<0.001). Furthermore, the median (Q1, Q3) lengths of stay increased from 0 (0.0, 4.0) to 2 (0.0, 4.0) and 4 (1.0, 6.0) days in the robust, pre-frail, and frail categories, respectively (p<0.001). The proportion of in-hospital deaths increased from 0.4% to 0.9% and 4.3% (p<0.001), home discharges decreased from 74% to 62% and 42% (p<0.001), and discharge to rehabilitation increased from 23% to 33% and 46% in the robust, pre-frail, and frail categories, respectively (p<0.001).

In the unadjusted models, being male, having two or more comorbidities, and having severe head injuries were associated with increased odds of trauma team activation (Table 3). Age, fall injuries, injury severity, having one or more comorbidities, and moderate and severe head injury were associated with increased odds of inpatient admission and decreased odds of ED discharge. Age, fall injuries, injury severity, one or more comorbidities, and severe head injury were associated with longer hospital stays (Table 4). Being Hispanic, having no health insurance, or being either underweight, overweight, or obese was associated with shorter hospital stays. Male sex, injury severity, and having three or more comorbidities were associated with in-hospital death. Being a non-Hispanic Black or Hispanic, having no health insurance or non-Medicaid/Medicare insurance, and being overweight or obese was associated with increased odds of home discharge. Age, fall injury, injury severity, and one or more comorbidities were associated with increased odds of discharge to rehabilitation.

**Table 3:** Unadjusted regression analysis assessing the relationship between frailty and measures of clinical care trajectory in the ED among older adults with trauma injuries (N = 2,862).

Variables	Trauma Team Activation	Inpatient Admission	ED Discharge
	Unadjusted MD (95% CI)**	Unadjusted OR (95% CI)*	Unadjusted OR (95% CI)*
Frailty Index			
Robust	Ref	Ref	Ref
Pre-Frail	**1.81 (1.49–2.21)**	**1.65 (1.39–1.96)**	**0.61 (0.52–0.73)**
Frail	**2.61 (2.10–3.25)**	**3.89 (3.12–4.86)**	**0.25 (0.20–0.32)**
Age in years (Mean (SD))	1.01 (0.99–1.01)	**1.05 (1.04–1.06)**	**0.95 (0.94–0.96)**
Sex*			
Female	Ref	Ref	Ref
Male	**1.92 (1.63–2.26)**	1.06 (0.90–1.24)	0.92 (0.79–1.08)
Race/Ethnicity			
Non-Hispanic Whites	Ref	Ref	Ref
Non-Hispanic Blacks	1.11 (0.71–1.73)	**0.66 (0.43–0.99)**	**1.55 (1.02–2.36)**
Hispanic	1.09 (0.88–1.36)	**0.56 (0.45–0.68)**	**1.79 (1.45–2.20)**
Other Races	0.99 (0.81–1.22)	0.96 (0.79–1.17)	1.02 (0.84–1.24)
Insurance Status			
Medicare/Medicaid	Ref	Ref	Ref
Other insurance	1.06 (0.90–1.24)	**0.79 (0.68–0.92)**	**1.24 (1.06–1.44)**
No insurance	1.58 (0.92–2.71)	**0.48 (0.28–0.81)**	**1.83 (1.08–3.09)**
Body Mass Index			
Normal	Ref	Ref	Ref
Underweight	**0.69 (0.48–0.99)**	1.33 (0.94–1.89)	0.75 (0.53–1.06)
Overweight	1.17 (0.96–1.41)	**0.61 (0.51–0.73)**	**1.65 (1.38–1.97)**
Obese	1.07 (0.86–1.33)	**0.58 (0.47–0.71)**	**1.71 (1.40–2.10)**
Injury type			
Non-fall injury	Ref	Ref	Ref
Fall-related injury	**0.42 (0.32–0.54)**	**1.64 (1.28–2.11)**	**0.67 (0.53–0.86)**
Recurrent fall-related visit			
No	Ref	Ref	Ref
Yes	0.99 (0.72–1.37)	1.28 (0.94–1.75)	0.80 (0.59–1.09)
Injury Severity	1.01 (0.99–1.03)	**1.34 (1.30–1.38)**	**0.74 (0.72–0.76)**
Charlson Comorbidity Index			
No comorbidity	Ref	Ref	Ref
1 comorbidity	1.14 (0.95–1.37)	**1.43 (1.21–1.70)**	**0.71 (0.60–0.84)**
2 comorbidities	**1.84 (1.45–2.32)**	**2.05 (1.62–2.61)**	**0.48 (0.38–0.62)**
3 or more comorbidities	**2.45 (1.81–3.38)**	**3.43 (2.36–4.97)**	**0.30 (0.21–0.44)**
Glasgow Coma Scale Score			
Mild	Ref	Ref	Ref
Moderate	3.20 (1.77–5.76)	**2.03 (1.03–4.02)**	**0.44 (0.22–0.89)**
Severe	**73.79 (10.04–541.97)**	**3.32 (1.27–8.68)**	**0.05 (0.01–0.39)**

OR: Odds ratio; Significant associations in bold

*Significant association interpreted as confidence interval not traversing one.

**Table 4:** Unadjusted regression analysis assessing the relationship between frailty and measures of hospital stay among older adults with trauma injuries (N = 2,862).

Variables	Length of Hospital Stay	Inhospital Death	Home Discharge	Discharge to Rehabilitation
	Unadjusted MD (95% CI)**	Unadjusted OR (95% CI)*	Unadjusted OR (95% CI)*	Unadjusted OR (95% CI)*
Frailty Index				
Robust	Ref	Ref	Ref	Ref
Pre-Frail	**2.00 (1.69–2.31)**	2.13 (0.67–6.70)	**0.56 (0.46–0.67)**	**1.68 (1.38–2.04)**
Frail	**4.00 (3.64–4.36)**	**10.48 (3.67–29.88)**	**0.25 (0.21–0.31)**	**2.99 (2.42–3.71)**
Age in years (Mean (SD))	**0.12 (0.10–0.13)**	1.02 (0.99–1.05)	**0.95 (0.94–0.96)**	**1.06 (1.05–1.07)**
Sex*				
Female	Ref	Ref	Ref	Ref
Male	0.00 (-0.56–0.56)	**2.76 (1.51–5.03)**	1.14 (0.97–1.33)	**0.70 (0.60–0.83)**
Race/Ethnicity				
Non-Hispanic Whites	Ref	Ref	Ref	Ref
Non-Hispanic Blacks	-1.00 (-2.06–0.06)	0.63 (0.08–4.65)	**2.77 (1.69–4.55)**	**0.41 (0.25–0.69)**
Hispanic	**-1.00 (-1.52 –-0.48)**	0.63 (0.24–1.62)	**2.01 (1.61–2.51)**	**0.47 (0.37–0.59)**
Other Races	0.00 (-0.48–0.48)	1.03 (0.50–2.12)	**1.58 (1.30–1.92)**	**0.61 (0.49–0.75)**
Insurance Status				
Medicare/Medicaid	Ref	Ref	Ref	Ref
Other insurance	0.00 (-0.40–0.40)	0.63 (0.33–1.19)	**1.30 (1.11–1.51)**	**0.73 (0.62–0.86)**
No insurance	**-2.00 (-3.40 –-0.60)**	2.97 (0.87–10.08)	**1.90 (1.06–3.41)**	**0.24 (0.11–0.53)**
Body Mass Index				
Normal	Ref	Ref	Ref	Ref
Underweight	**-1.00 (-1.87 –-0.13)**	1.58 (0.44–5.65)	0.94 (0.69–1.29)	0.96 (0.69–1.32)
Overweight	**-2.00 (-2.48 –-1.51)**	2.01 (0.97–4.16)	**1.46 (1.22–1.75)**	**0.64 (0.53–0.78)**
Obese	**-2.00 (-2.55 –-1.45)**	1.81 (0.80–4.13)	**1.43 (1.16–1.75)**	**0.66 (0.53–0.81)**
Injury type				
Non-fall injury	Ref	Ref	Ref	Ref
Fall-related injury	**1.00 (0.14–1.86)**	0.86 (0.34–2.20)	**0.71 (0.55–0.93)**	**1.79 (1.33–2.40)**
Recurrent fall-related visit				
No	Ref	Ref	Ref	Ref
Yes	0.00 (-1.00–1.00)	0.65 (0.16–2.71)	0.93 (0.69–1.25)	1.16 (0.85–1.58)
Injury Severity	**0.38 (0.35–0.40)**	**1.10 (1.07–1.14)**	**0.88 (0.86–0.89)**	**1.10 (1.08–1.12)**
Charlson Comorbidity Index				
No comorbidity	Ref	Ref	Ref	Ref
1 comorbidity	**1.00 (0.41–1.59)**	1.54 (0.75–3.20)	**0.80 (0.67–0.95)**	**1.27 (1.06–1.52)**
2 comorbidities	**2.00 (1.21–2.79)**	1.59 (0.63–4.01)	**0.65 (0.52–0.81)**	**1.45 (1.15–1.84)**
3 or more comorbidities	**3.00 (1.92–4.08)**	**4.03 (1.65–9.86)**	**0.40 (0.29–0.55)**	**1.94 (1.42–2.64)**
Glasgow Coma Scale Score				
Mild	Ref	Ref	Ref	Ref
Moderate	2.00 (-0.11–4.11)	**9.38 (3.15–27.92)**	**0.27 (0.14–0.50)**	1.45 (0.80–2.62)
Severe	**3.00 (0.43–5.57)**	**71.11 (31.80–159.01)**	**0.12 (0.04–0.31)**	0.49 (0.20–1.21)

OR: Odds ratio; MD: Median Difference

*Significant association in bold = confidence interval not traversing one

**Significant association in bold = confidence interval not traversing zero

After adjusting for the potential confounders, pre-injury frailty was significantly associated with the measures of clinical care trajectory (Table 5). Compared to those categorized as robust, patients categorized as pre-frail (AOR: 2.04; 95% CI: 1.63–2.54) and frail (AOR: 2.45; 95% CI: 1.86–3.23) had two times the odds of having trauma teams activated for their care. Compared to those categorized as robust, patients categorized as pre-frail and frail had 1.7 (95% CI: 1.34–2.07) and 3.1 (95% CI: 2.3–4.1) times the adjusted odds of inpatient admission and 42% (AOR: 0.58; 95% CI: 0.47–0.73) and 69% (AOR: 0.31; 95% CI: 0.23–0.42) reduced odds of ED discharge, respectively. Compared to those categorized as robust, patients categorized as frail had a 1.4 (95% CI: 0.97–1.75) adjusted median increase in their lengths of hospital stays and 3.7 (95% CI: 1.07–12.62) times the odds of dying while on admission. Compared to those categorized as robust, patients categorized as pre-frail and frail had 37% (AOR: 0.63; 95% CI: 0.51–0.78) and 64% (AOR: 0.36; 95% CI: 0.28–0.47) reduced odds of home discharge, respectively. Compared to those categorized as robust, patients categorized as pre-frail and frail had 1.4 (95% CI: 1.12–1.73) and 2.2 (95% CI: 1.71–2.91) times the adjusted odds of discharge to rehabilitation, respectively.

**Table 5:** Adjusted odds and median difference in the measures of care trajectory among older adults with trauma injuries across the frailty spectrum (N = 2,862).

Outcome Variables	Predictor Variable: Frailty	
	Pre-Frail	Frail
	Adjusted Odds Ratio (95% CI)	Adjusted Odds Ratio (95% CI)
Trauma team activated	**2.04 (1.63–2.54)**	**2.45 (1.86–3.23)**
Inpatient admission	**1.67 (1.34–2.07)**	**3.06 (2.28–4.12)**
ED Discharge	**0.58 (0.47–0.73)**	**0.31 (0.23–0.42)**
Length of stay	0.25 (-0.04–0.56)	**1.36 (0.97–1.75)**
Inhospital death	1.66 (0.50–5.45)	**3.68 (1.07–12.62)**
Home discharge	**0.63 (0.51–0.78)**	**0.36 (0.28–0.47)**
Discharge to rehabilitation	**1.39 (1.12–1.73)**	**2.23 (1.71–2.91)**

Each row represents a single model, with the outcome variables of each model listed in the leftmost column, while the predictor variable is frailty, measured as a three-level categorical variable–robust, pre-frail, and frail. Robust category is the reference category in all the models and it is not depicted in this table. Each model controlled for age, sex, race/ethnicity, insurance type, body mass index, injury type, recurrent fall injury, injury severity, Charlson comorbidity index, and Glasgow Coma Scale score

**Fig 1:**
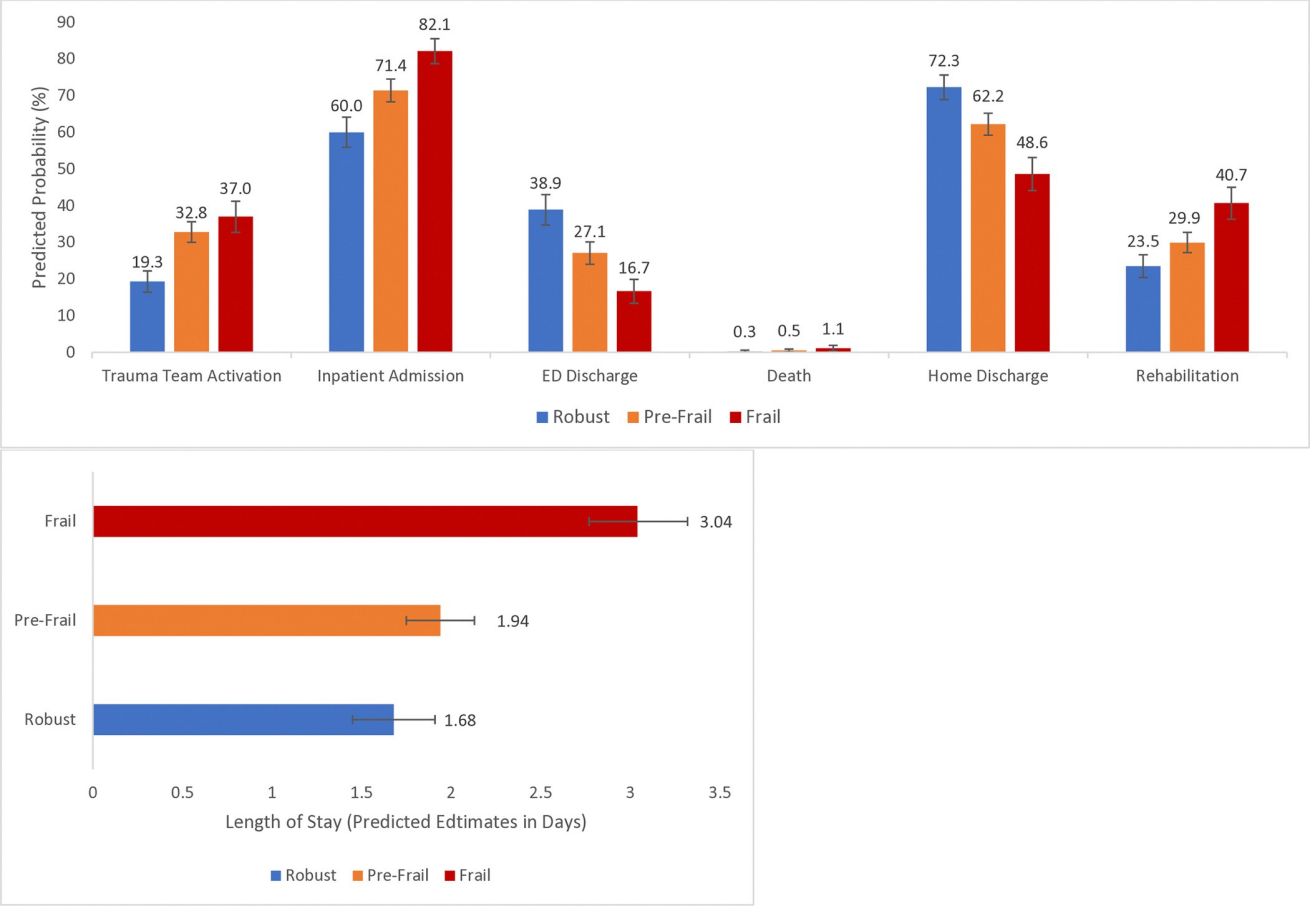
Predicted probabilities of inpatient admission, home discharge, discharge to rehabilitation, and the predicted estimates of the lengths of hospital stay across the robust, pre-frail, and frail spectrum among older adults with trauma injuries (N = 2,862); Each model controlled for age, sex, race/ethnicity, insurance type, body mass index, injury mechanism, recurrent fall-related visit, injury severity, Charlson comorbidity index, and Glasgow Coma Scale score.

The predicted probability of trauma team activation increased from 19% (95% CI: 16.4–22.2), to 33% (95% CI: 30.0–35.6), and 37% (95% CI: 32.7–41.2) in the robust, pre-frail, and frail categories, respectively (Fig 1). Also, the predicted probability of inpatient admission increased from 60% (95% CI: 55.9–64.1) to 71% (95% CI: 68.3–74.5) and 82% (95% CI: 78.7–85.5) across the robust, pre-frail, and frail categories. Conversely, the predicted probabilities of ED discharge reduced from 39% (95% CI: 34.7–43.0) to 27% (95% CI: 24.0–30.1) and 17% (95% CI: 13.4–19.9) in the robust, pre-frail, and frail categories, respectively. The predicted median lengths of hospital stay were 1.7 days (95% CI: 1.45–1.91) in the robust category and 3.0 days (95% CI: 2.77–3.32) in the frail category. The predicted probability of in-hospital death was 0.3% (0.0–0.6) in the robust category and 1.1% (95% CI: 0.3–1.9) in the frail category. As the predicted probabilities of home discharge reduced from 72% (95% CI: 68.9–75.6) to 62% (95% CI: 59.2–65.2) and 49% (95% CI: 44.1–53.1) across the robust, pre-frail, and frail categories, the predicted probabilities of discharge to rehabilitation increased from 24% (95% CI: 20.4–26.6) to 30% (95% CI: 27.2–32.7) and 41% (95% CI: 36.3–45.0) across the robust, pre-frail, and frail, categories, respectively.

## Discussion

To our knowledge, this is one of the few studies that report the association between pre-injury frailty and the clinical care trajectory of older adults with trauma injuries. Earlier studies have reported that frailty increases the odds of inpatient admission, 30-day re-presentation in the ED, and mortality due to an inability to mount up adequate physiologic response to injuries and diseases [31–33]. Our study builds on this knowledge by showing that frail older adults with trauma injuries are more likely to have trauma team activation upon presentation, be admitted from the ED, experience longer hospital stays, and either die or be discharged to rehabilitation during their index hospital admission. We also report the dose-response pattern in the predicted odds and estimates of these measures of clinical care trajectories across the robust, pre-frail, and frail categories.

We found that frailty is associated with higher levels of trauma team activation at ED presentation. Earlier research on frail older adults has indicated that frailty often leads to more severe clinical presentations and worse outcomes after injury [34, 35], which may explain the increased trauma activations. In this context, trauma teams might be more inclined to escalate care due to the greater likelihood of complications in frail patients, even when their injuries appear less severe compared to non-frail individuals [36, 37]. This pattern may reflect a heightened precautionary approach to managing frail patients, who are more vulnerable to rapid deterioration.

Our study reports that when frail older adults present with trauma injuries, they are more likely to be admitted and less likely to be discharged from the ED. This increased likelihood of inpatient admissions and fewer discharges from the ED among frail older adults may be attributed to the complex, multi-system health issues that often accompany frailty [38, 39]. Frail older adults are likely to have multiple coexisting conditions (e.g., cardiovascular or respiratory problems) that complicate their recovery and necessitate more prolonged observation or treatment, even for relatively minor injuries [40–42]. Moreover, frail older adults may require more time and specialized care to recover their functional independence [43, 44], making immediate ED discharge less feasible. Health system protocols that prioritize safety in frail populations may also drive this association, as clinicians err on the side of caution to prevent adverse outcomes like readmission or deterioration after discharge [45, 46].

Earlier studies have assessed the relationship between frailty and length of hospital stay among older adults with trauma injuries and have provided conflicting results. While studies conducted in developed countries like the United Kingdom, Germany, and Sweden have reported no difference in lengths of hospital stay [19, 47], US-based studies have consistently reported longer lengths of hospital stay for those who are frail [21, 48, 49]. This difference may be a reflection of different health system policies across countries. Studies outside the US report a mean length of hospital stay of 16 to 20 days [19, 47]. Conversely, the length of hospital stay among US older adults with trauma injuries is three days for non-surgical patients and seven to nine days for those who have surgery [21, 48, 49]. Additionally, one of Medicare policies, the primary payer of health coverage for older adults, that governs admission into acute rehabilitation facilities is the three-day rule, which requires an inpatient stay of at least three days, excluding the day of admission [50]. Thus, the three-day rule may explain the pattern of the median stay among patients identified as frail in this study.

We found that frailty was associated with increased odds of in-hospital deaths. This finding aligns with existing literature that identifies frailty as a strong predictor of mortality among older adults [51–54]. Older adults who are frail and subsequently admitted to the hospital are more vulnerable to complications like infections, organ failure, and delayed recovery, all of which can increase the risk of death [55, 56]. Older adults with frailty may not always benefit from aggressive life-saving interventions in the same way as non-frail older adults. The weakened state of older adults with frailty could render them less capable of withstanding invasive procedures or prolonged intensive care, ultimately leading to worse outcomes despite heightened efforts to save their lives. Furthermore, the presence of advanced comorbidities common in older adults with frailty can further complicate their clinical trajectory, increasing the likelihood of fatal outcomes during hospitalization.

Our study shows that older adults with frailty who sustained trauma injuries were less likely to be discharged home but were more likely to be discharged to rehabilitation centers such as acute or subacute care rehabilitation centers or skilled nursing facilities. Discharge to rehabilitation centers has its benefits, some of which include access to multidisciplinary care, reduction in unnecessary ED re-presentation and readmission, and access to physical therapy [57]. Such discharge disposition is, therefore, not a negative disposition but a less preferred option to home discharge. The option of discharging to acute or subacute care rehabilitation centers and skilled nursing facilities will be further deprecated if older adults with trauma injuries lose one or more domains of activities of daily living during their hospital stay. Disregarding inpatient frailty progression on the assumption that functions lost would be regained or managed in rehabilitation centers is suboptimal care and must be avoided. With skeletal atrophy setting in within 72 hours of immobility [15, 16], care plans of older adults with trauma injuries should include evidence-based interventions such as comprehensive geriatric assessment, exercise and early ambulation, nutritional rehabilitation, and avoidance or limiting the use of tethering devices such as intravenous lines and catheters [19, 58].

This study has its limitations. Three of the components of the FRAIL index (fatigue, resistance, and ambulation) are self-reported measures. Self-reported bias, therefore, cannot be excluded. Although the FRAIL index has demonstrated strong validity, it has weak reliability. Hence, there is a likelihood that when administered under the same condition, responses of older adults with trauma injuries may vary. The statistically significant difference we report between the pre-injury frailty and the lengths of hospital stay may lack clinical relevance since the median difference was approximately one day across each category. Our results may, however, have greater relevance to a smaller subset of older adults with trauma injuries with extended hospital stays (the right-skewed population). Interventions aimed at reducing frailty progression among admitted older adults with trauma injuries must, therefore, be focused on identifying those likely to have longer stays. Despite these limitations, this study is the first to demonstrate the dose-response patterns in the ED and hospital discharge dispositions of older adults with trauma injuries across the frailty spectrum. Future studies should explore the extent to which evidence-based interventions impact frailty progression among admitted older adults with trauma injuries.

The findings of this study have significant implications. Our identification of a dose-response pattern in the ED and hospital discharge dispositions across frailty categories underscores the importance of recognizing frailty as a spectrum that can be managed. Recognizing that pre-injury frailty may worsen in the setting of trauma injury can guide clinicians in anticipating and managing the clinical care needs of frail individuals, potentially mitigating adverse outcomes and improving patient-centered care delivery. Such care needs may include early mobilization strategies to prevent functional decline, comprehensive geriatric assessments to identify and address underlying medical and psychosocial issues, multidisciplinary rehabilitation programs tailored to individual frailty levels, and proactive discharge planning to facilitate safe transitions to appropriate post-acute care settings. Moreover, integrating patient and caregiver education initiatives into the care continuum can empower individuals and their families to actively participate in decision-making processes and self-management strategies, thereby promoting autonomy and enhancing overall quality of life. By addressing these multifaceted care needs within a patient-centered framework, healthcare providers can strive to optimize outcomes and foster resilience in frail older adults following trauma injury.

## Conclusion

Pre-injury frailty is associated with an increased likelihood of trauma team activation, inpatient admission from the ED, prolonged length of hospital stay, reduced discharges to home, increased discharge to rehabilitation, and increased odds of in-hospital death among older adults with trauma injuries. Indeed, it is impossible to correct pre-injury frailty during a trauma admission. However, a lot can be done to slow down or reduce frailty progression through multidisciplinary care shared by the trauma, geriatric, emergency medicine, physiotherapy, social work, case management and nutritional teams. Screening for pre-injury frailty, early and continued inpatient ambulation, and nutritional rehabilitation may slow down frailty progression and improve the quality of care for older adults with trauma injuries.
